# Megakaryocytes in Peripheral Blood Smears

**DOI:** 10.4274/tjh.galenos.2019.2019.0022

**Published:** 2019-08-02

**Authors:** Neha Garg, Rashmi Jain Gupta, Sunil Kumar

**Affiliations:** 1 Lok Nayak Hospital, Department of Clinical Pathology, Delhi Gate, New Delhi, India

**Keywords:** Megakaryocyte, Peripheral blood smear, Peripheral blood

## To the Editor,

Megakaryocytes (MKs) are large polypoidal cells found within the bone marrow (BM), comprising 0.01% of all nucleated cells [1]. Circulating MKs have been described in the literature but normal MKs in peripheral blood smears (PBSs) have rarely been reported [2]. We report here 4 cases in which we found MKs in the PBS.

**Figure 1:**
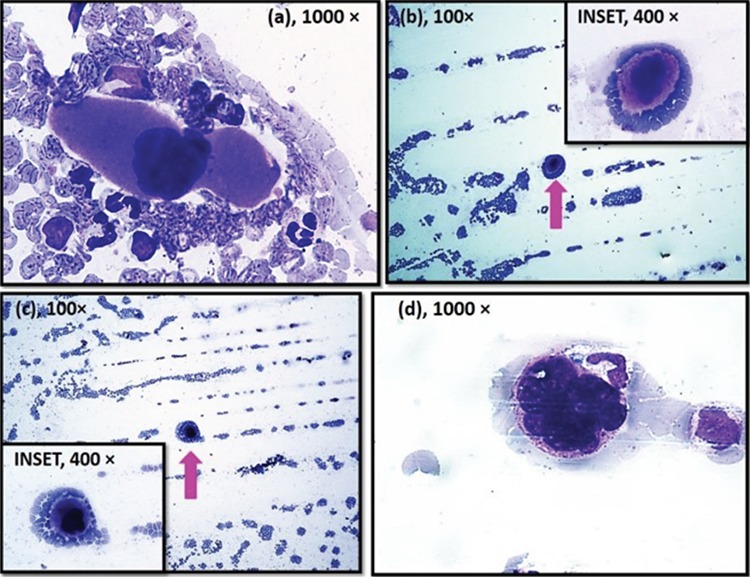
(a-d) High power view of Giemsa-stained peripheral blood smear (PBS) in cases 1-4, respectively, showing megakaryocyte, round-elongated in shape, with moderate-abundant amount of granular cytoplasm with compact lobulated nucleus at the tail end of the PBS. (b, c) A large megakaryocyte is seen at the tail end of the PBS in cases 2 and 3 (100^x^).

In the first case, a 10-year-old boy presented with weakness and decreased appetite. PBS showed microcytic hypochromic anemia (MHA), leukocytosis, and reactive thrombocytosis. In the second case, a 30-year-old female presented with fever and skin rash with positive dengue serology. PBS showed MHA with thrombocytopenia. In the third case, a 15-year-old female presented with fever with chills and rigor. PBS showed macrocytic anemia, thrombocytopenia, and trophozoites of *Plasmodium vivax*. In the fourth case, a 14-year-old male was admitted for grafting for a burn on his hand. PBS showed MHA with reactive thrombocytosis. A MK, round-elongated in shape, with moderate-abundant amount of granular cytoplasm and compact lobulated nucleus, was seen in each of these cases at the tail end of the PBS (Figures 1a-1d). None of these patients had hepatosplenomegaly or evidence of any hematological disorders. Table 1 shows their hematological parameters.

MKs develop from hematopoietic stem cells that reside in the BM. The finding of MKs in peripheral blood (PB) is usually indicative of a serious disorder of the BM, such as myelodysplasia, granulocytic leukemia, or other myeloproliferative disorders [3]. PBSs in such cases may show leukoerythroblastic reaction, large cytoplasmic fragments of MKs, and dwarf micromegakaryocytes [3]. Though normal MKs were reported in PBS in a case of post-essential thrombocythemia-myelofibrosis, the PBS additionally showed the presence of leukoerythroblastic reaction and blasts [2]. Our patients had neither a history of myeloproliferative disorders nor such additional findings in the PBS.

In 1965 Kaufman et al. [4] demonstrated that 20%-25% of mature MKs leave the BM with sufficient cytoplasm to enter the PB and migrate to the lungs and 7%-17% of the body’s platelets are released in pulmonary capillaries, hence suggesting that MKs normally circulate in the PB and are normal constituents of the PB [4]. However, the identification of normal MKs in PBSs of normal patients has never been reported.

In response to anemia, there is stimulation of erythropoietin (EPO) receptors by EPO, present on erythroid precursors as well as on MKs in BM [5]. There is also an increase in TPO levels in response to thrombocytopenia, with resultant increase in MK differentiation. This might be a cause of increase in number of circulating MKs with consequent detection in the PBS.

In conclusion, detection of MKs in PB is usually indicative of a serious BM disturbance. However, one should keep in mind that mature MKs normally circulate in the PB and can also be seen in PBSs in cases of increased MK differentiation. The clinical significance of this finding is unclear, but as large cells like blasts, large atypical lymphocytes, organisms, or in this case MKs can be found at the edge of PBSs, a routine examination of the feathered edge of the PBS is advised as it has diagnostic importance.

## Figures and Tables

**Table 1:**
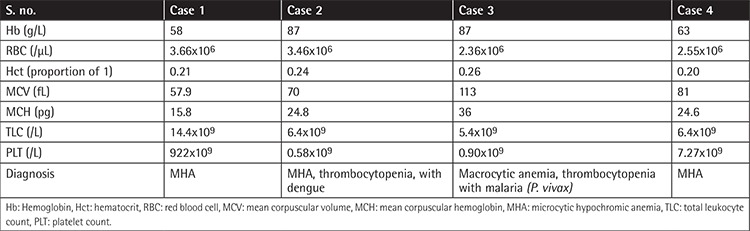
Hematological parameters with diagnosis.
